# Diffusion of sylvatic yellow fever in the state of São Paulo, Brazil

**DOI:** 10.1038/s41598-021-95539-w

**Published:** 2021-08-11

**Authors:** Alec Brian Lacerda, Leila del Castillo Saad, Priscilla Venâncio Ikefuti, Adriano Pinter, Francisco Chiaravalloti-Neto

**Affiliations:** 1 Department of Epidemiology, School of Public Health-University of Sao Paulo, Av. Dr. Arnaldo, 715, São Paulo, SP Brazil; 2 Epidemiological Surveillance Center “Prof. Alexandre Vranjac” of the Health Secretariat of the State of São Paulo, São Paulo, SP Brazil; 3 SUCEN, Endemics Control Superintendence, São Paulo, SP Brazil

**Keywords:** Diseases, Ecological epidemiology

## Abstract

We investigated the sylvatic yellow fever (SYF) diffusion process in São Paulo (SP) between 2016 and 2019. We developed an ecological study of SYF through autochthonous human cases and epizootics of non-human primates (NHPs) that were spatiotemporally evaluated. We used kriging to obtain maps with isochrones representative of the evolution of the outbreak and characterized its diffusion pattern. We confirmed 648 human cases of SYF in SP, with 230 deaths and 843 NHP epizootics. Two outbreak waves were identified: one from West to East (2016 and 2017), and another from the Campinas region to the municipalities bordering Rio de Janeiro, Minas Gerais, and Paraná and those of the SP coast (2017–2019). The SYF outbreak diffusion process was by contagion. The disease did not exhibit jumps between municipalities, indicating that the mosquitoes and NHPs were responsible for transmitting the virus. There were not enough vaccines to meet the population at risk; hence, health authorities used information about the epizootic occurrence in NHPs in forest fragments to identify priority populations for vaccination.

## Introduction

Yellow fever (YF) is a non-contagious viral disease of short duration, presenting with mild and, in some cases, severe symptoms that may cause death. YF is caused by a *Flavivirus* of the family Flaviviridae, and it is divided into two transmission cycles: sylvatic and urban, with similar etiological, pathophysiological, and clinical aspects^1^. In the urban cycle, the YF virus is maintained and transmitted by *Aedes aegypti* mosquito^2^, which directly infects humans that become an amplifier and infection source for new mosquitoes in urban areas. This cycle is nowadays reported in parts of Africa and can be blocked by increase the vaccination coverage, since human being is part of the cycle. In the sylvatic cycle, the disease is a zoonosis transmitted mainly by the vectors *Haemagogus* sp and *Sabethes* sp mosquitoes (in Brazil, *Haemagogus leucocelanos* and *Haemagogus janthinomys*^1,3^), which infect non-human primates (NHPs) that become an amplifier and infection source for new mosquitos. This occurs particularly with primates of the genera *Cebus*, *Alouatta*, and *Callithrix* that are very sensitive to the infection and were very often found dead because of the disease^2,4,5^. In this cycle, unvaccinated humans are accidentally infected when they enter or live nearby the habitat of the infected mosquito vector, usually inside or around remained forest sites. This cycle is reported in Brazil, and the increase of vaccination coverage is aimed to protect the human population but cannot blocks the transmission and propagation, hence it keeps circulating among NHPs.

The process of infection diffusion can be characterized as its spread over time, constituting a space–time phenomenon that can occur by expansion, relocation, or a hybrid characteristic^6,7^. Diffusion by expansion represents dispersion in space–time, leaving one or a few points to new, located in its vicinity, expanding. This type of diffusion may be due to (1) contagion, wherein individuals in close proximity are more likely to have contact than distanced individuals, and (2) hierarchical diffusion, which begins in a large area then spreads to medium or small areas^7,8^. In relocation diffusion, an infection agent leaves its original zone and moves to a different (usually more favorable) location, such as during migration. In hybrid diffusion, relocation occurs, but the center is still active and expanding^6^.

**Figure 1:**
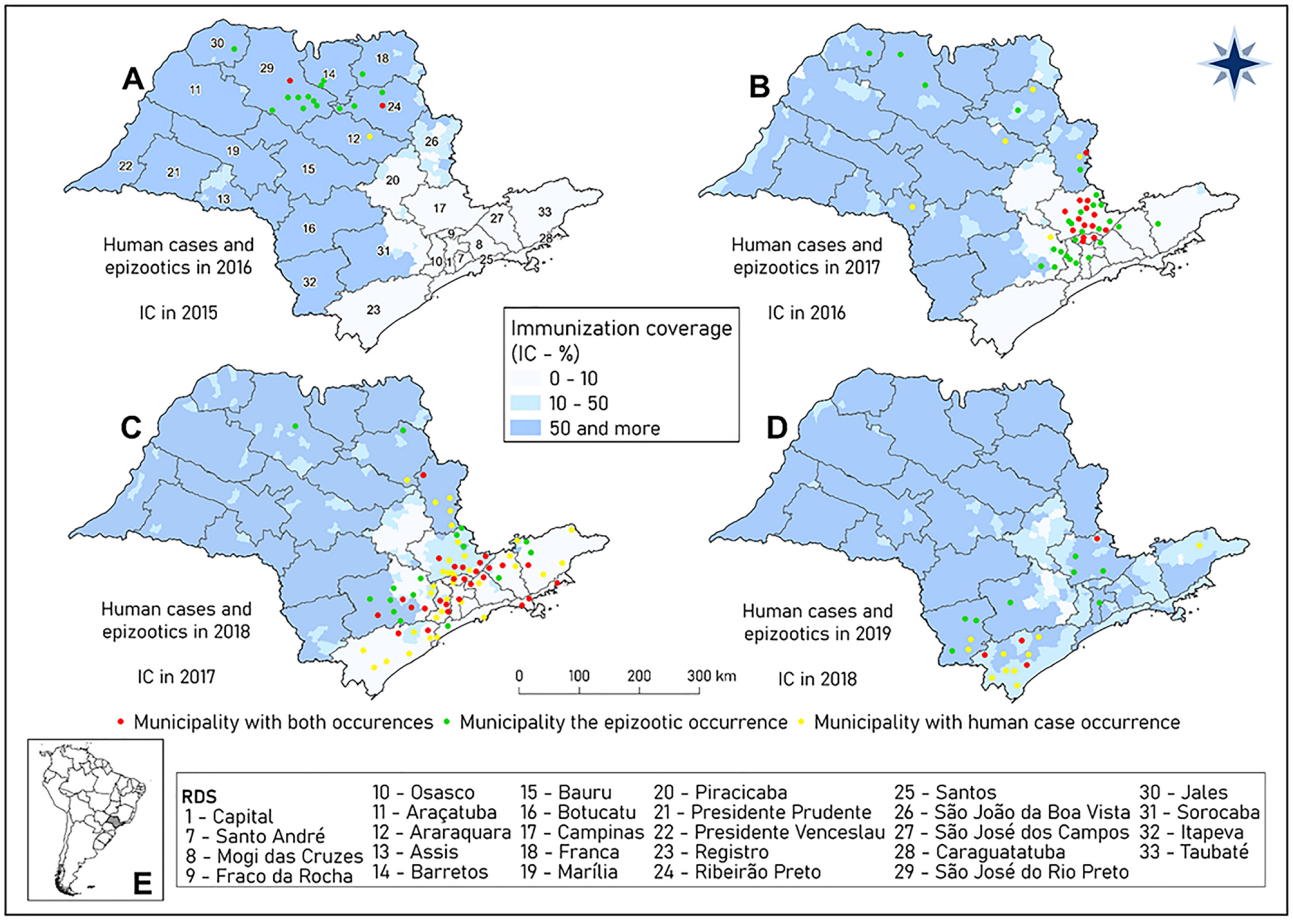
Maps of municipalities and Regional Disease Surveillance (RDS) with the occurrence of yellow fever (human autochthonous cases, epizootics and both) in 2016 (**A**), 2017 (**B**), 2018 (**C**) and 2019 (**D**) and, respectively, the vaccination coverages in the immediately preceding year, state of São Paulo. Map (**E**) presents the localization of the state of São Paulo in Brazil and South America. Maps created using the Free and Open Source QGIS.

The last major outbreak of urban yellow fever (UYF) in Brazil occurred in the city of Rio de Janeiro in 1929. Nevertheless, the last cases were recorded in 1942 in the state of Acre, in the city of Sena Madureira^9^. Until 1999, the sylvatic disease was restricted to the northern and central parts of the country, with sporadic cases in the southeast region. Since the 2000s, the state of São Paulo (SP) (Fig. 1) has been one of the centers of virus expansion and circulation in Brazil. In 2000, SP was the target of the first detection of autochthonous cases of sylvatic yellow fever (SYF) in the northwest region^2,10^. In 2008, the municipality of Ribeirão Preto confirmed human autochthonous cases and deaths. Simultaneously, cases of YF in NHPs were detected in the region of São José do Rio Preto. The year of 2009 was characterized by outbreaks, with human cases and deaths occurring in the south-central region of the state. Therefore, the area of SP with a vaccine recommendation against YF has been expanding since 2000^10^. From 2009 until 2015, 455 municipalities (approximately 10 million people, 25% of the SP state population) among 645 had a vaccine recommendation against the virus^11^.

In 2016, a new outbreak cycle was detected in SP, with the occurrence of autochthonous human cases and SYF epizootics in municipalities within the northwest and northern regions of the state^4,12,13^. In subsequent years, this cycle underwent a diffusion process, reaching municipalities located in the east, south, southeast, and northeast regions, which had low vaccination coverage levels and/or did not have vaccination recommendations^12^. The risk areas expanded within a short period of time, increasing the target population that should receive the vaccine from 10 to 30 million. In these areas with a large population, a large number of human cases and deaths due to YF has been observed, generating health crises and putting pressure on health units as people searched for vaccines^12,14^. To the extent that there was insufficient time and vaccine doses available to serve the entire population of the expanded areas at risk, the State Department of Health of São Paulo (SES-SP) used information on the epizootic occurrence among NHPs to predict spatial and temporal propagation through the fragmented forest. This approach was used to classify the priority populations to receive the vaccine at least two months prior to the onset of YF virus circulation in the area^15^. This strategy currently recognized by the Ministry of Health^16^ differed from the previous strategy, which recommended the assessment of the need for vaccination in a given municipality considering it as an indivisible unit^17^. Moreover, this current strategy has been adopted at the federal level and is being used following the epizootic propagation of the virus through the southern part of the country during the 2020/2021 season^18^.

The aim of this study was to describe and characterize the process of YF diffusion in SP between 2016 and 2019. We intend to broaden our understanding of this outbreak and contribute to the expansion of knowledge about disease surveillance and control. The identification of the diffusion type involved in this outbreak allows, for example, a discussion on the extent to which the vaccination strategy adopted by SES-SP was relevant. Understanding how the process of YF diffusion in SP occurred may be useful for future preventive action, possibly eliminating or mitigating health damage in vulnerable populations.

## Results

**Figure 2:**
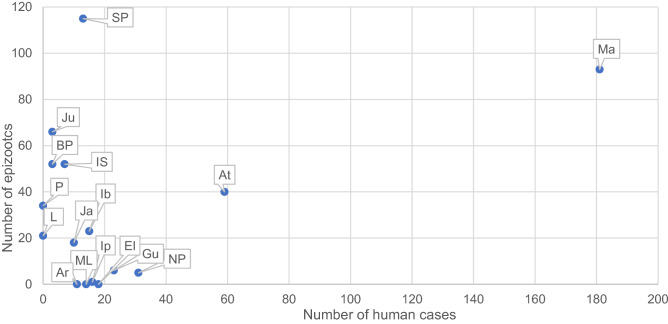
Ten municipalities of the state of São Paulo with more autochthonous human cases of yellow fever and ten with more epizootics between 2016 and 2019 (*Ma* Mairiporã, *At* Atibaia, *NP* Nazaré Paulista, *Gu* Guarulhos, *El* Eldorado, *Ip* Iporanga, *Ib* Ibiúna, *ML* Monteiro Lobato, *SP* São Paulo, *Ar* Arujá, *Ju* Junidaí, *BP* Bragança Paulista, *IS* Itapecerica da Serra, *Pi* Pinhalzinho, *Lo* Louveira, *Ja* Janiru).

From 2016 to 2019, 648 autochthonous human cases of SYF were confirmed in SP, with 230 deaths (fatality rate: 35.5%) and 843 NHP epizootics. The human cases and epizootics were detected in 91 and 89 municipalities, respectively. Ten of them were responsible for 58.8% of the human cases (381), with emphasis on Mairiporã (181 cases, 27.9%) and Atibaia (59 cases, 9.1%). Ten municipalities accounted for 64.2% of the NHP epizootics (541), with an emphasis on São Paulo (115 epizootics, 13.6%) and Mairiporã (93 epizootics, 11.0%). Figure 2 shows the dispersion diagram and the relationship among these municipalities, wherein four situations can be identified: (1) Mairiporã (1st in human cases and 2nd in epizootics) and Atibaia (2nd in human cases and 6th in epizootics) presented high numbers of human cases and epizootics; (2) Nazaré Paulista, Guarulhos, Eldorado, Iporanga, Monteiro Lobato, and Arujá had intermediate numbers of human cases and few or no epizootics; (3) São Paulo and Ibiúna presented an intermediate number of cases and a high number of epizootics; and (4) Jundiaí, Bragança Paulista, Itapecerica da Serra, Pinhalzinho, and Louveira, had few or no human cases, but a high number of epizootics.

**Figure 3:**
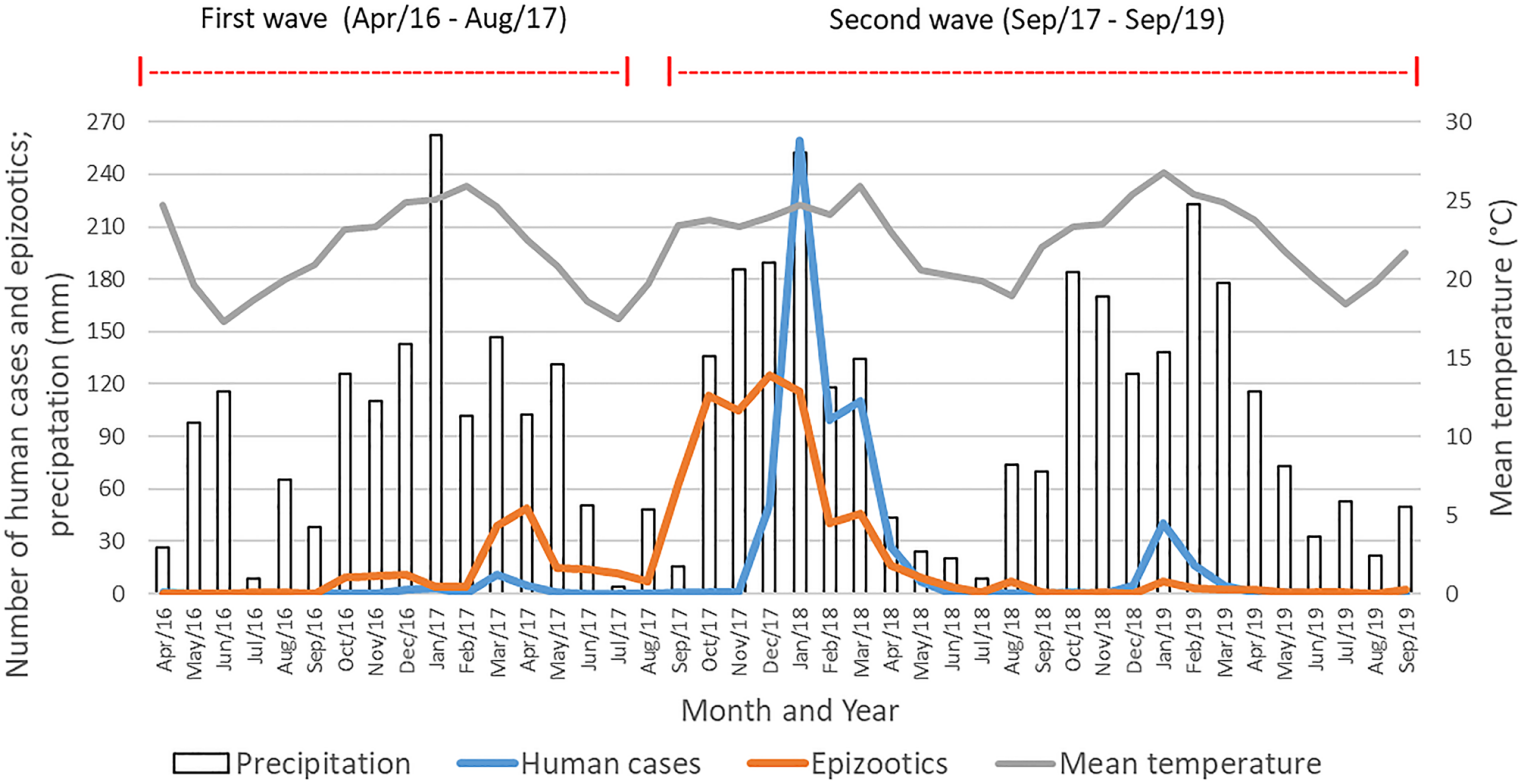
Autochthonous human cases, per month of symptom onset, and epizootics, per month of notification, of yellow fever and the two outbreak waves; precipitation and mean temperature; state of São Paulo, 2016 and 2019.

**Figure 4:**
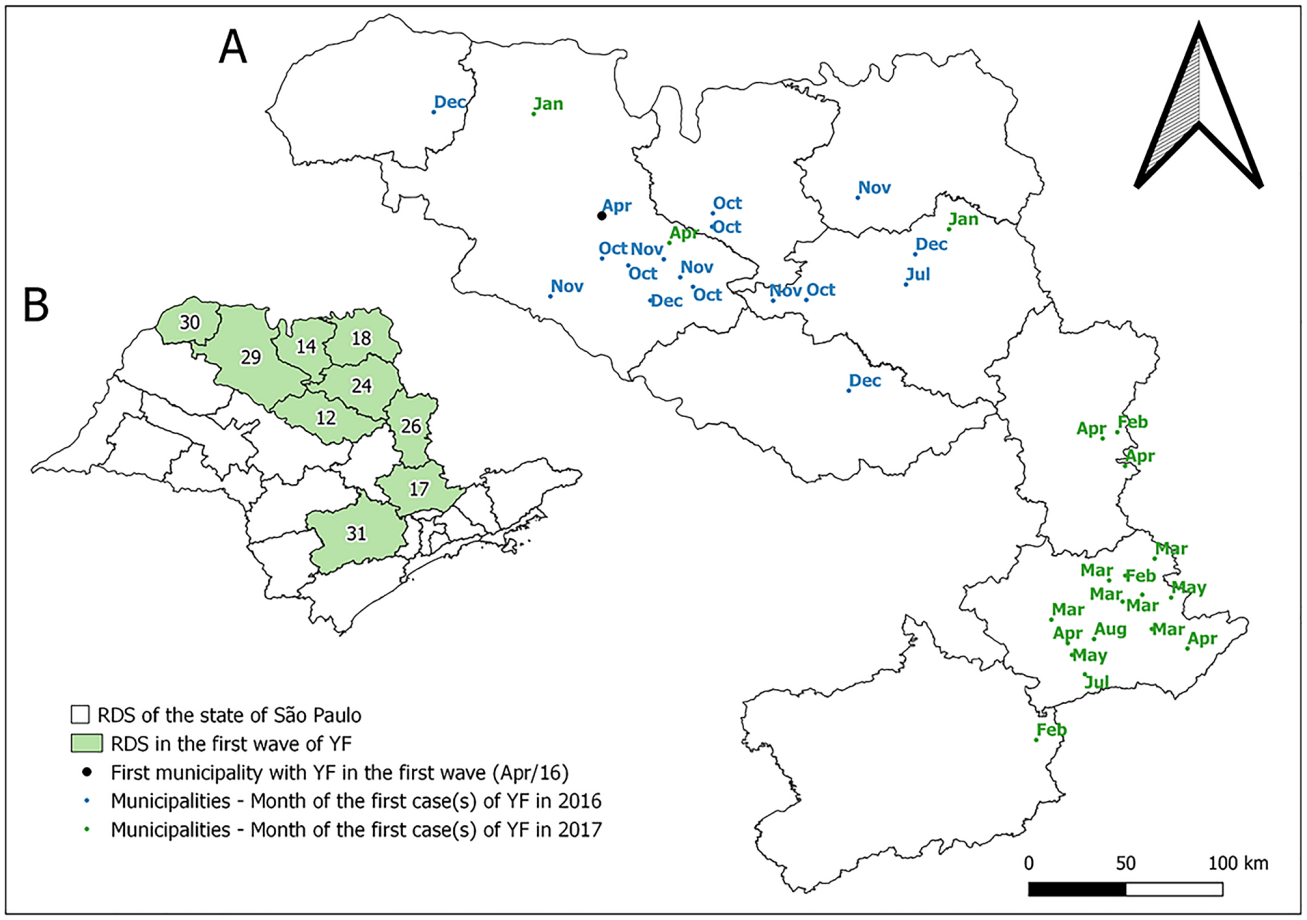
Map (**A**) with the date (month and year) of the first occurrences of yellow fever (YF) (autochthonous human cases, epizootics and both) in the municipalities of the state of São Paulo during the first outbreak wave, April 2016 to August 2017. Map (**B**) presents the Regional Disease Surveillance affected. Maps created using the Free and Open Source QGIS.

Figure 3 shows the temporal distribution of the occurrence of human cases and epizootics and precipitation and mean temperature values of SP between April 2016 and September 2019. The first autochthonous SYF occurrence during the study period was in April 2016, in a human residing in Bady Bassit, with São José do Rio Preto as the probable infection site, both of which are under the Regional Disease Surveillance (RDS) of São José do Rio Preto. The first two detected epizootics were in Ribeirão Preto, in the RDS of the same name, and in São José do Rio Preto, in July and August 2016, respectively (Figs. 1A, 4). Subsequently, between October 2016 and August 2017, the SYF autochthonous human cases and epizootics advanced from the western region of SP (high vaccination coverage) to the eastern region, particularly the RDS of Campinas (low vaccination coverage) (Fig. 1A,B, 4). An increase in epizootic diseases was detected in October 2016, reaching its peak in April, and then decreasing until August, following the increase and decrease of precipitation and temperature that occurred in the same period.

As for human cases, new cases were detected in December 2016 after the first occurrence in April 2016. After reaching its peak in March, a decrease occurred until August 2017 (Fig. 3). Notably, epizootic disease occurred two months earlier than human cases. The period between April 2016 and August 2017 was considered in this study as the first SYF wave in SP, given that it included the first disease occurrences, its first peaks, and the first moment of decrease in cases and epizootics (Fig. 3). This definition also considered the arrival of the outbreak process in the eastern SP region, which had low vaccination coverage (Figs. 1, 4).

**Figure 5:**
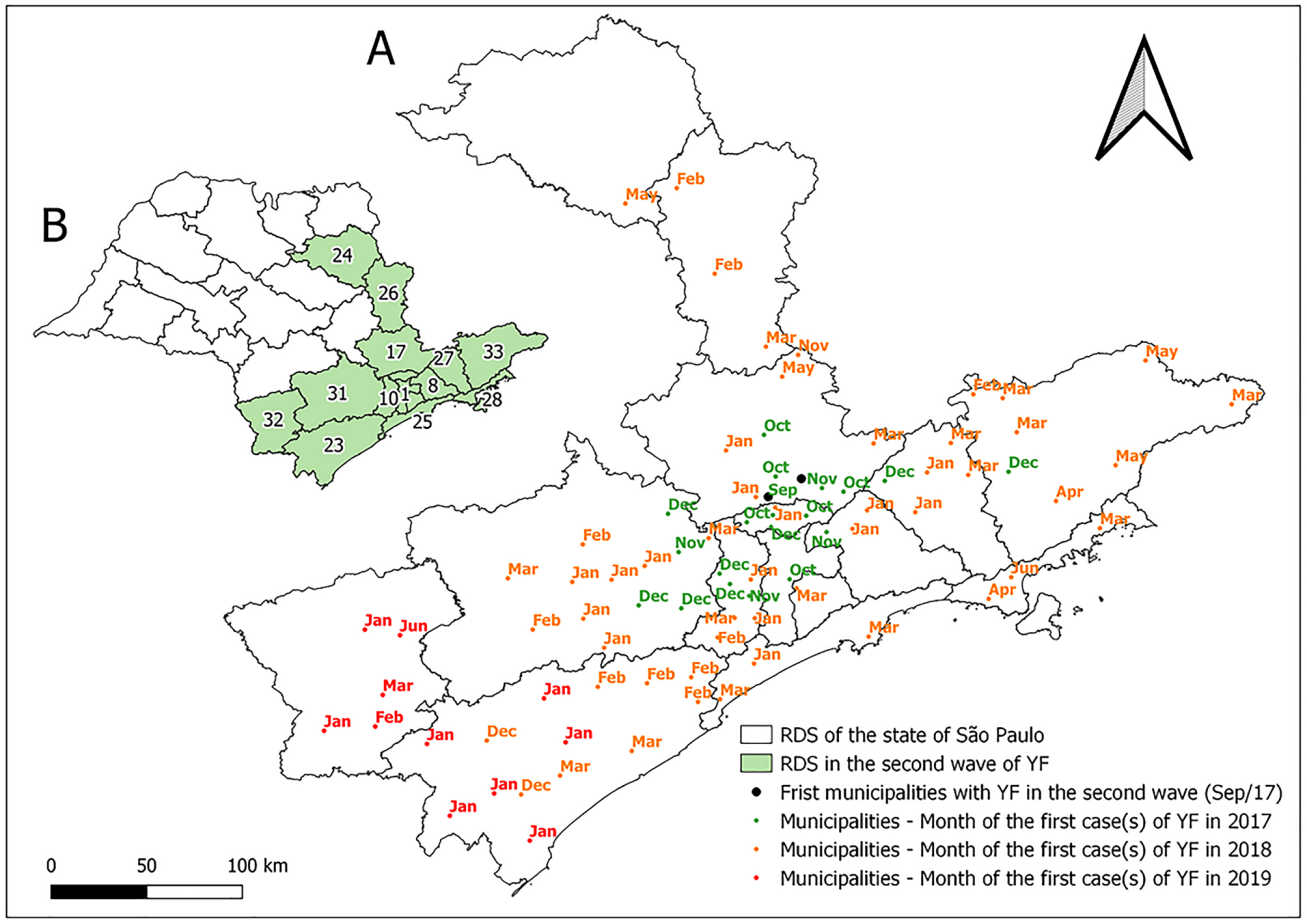
Map (**A**) with the date (month and year) of the first occurrences of yellow fever (YF) (autochthonous human cases, epizootics and both) in the municipalities of the state of São Paulo during the second outbreak wave, September 2016 to September 2017. Map (**B**) presents the Regional Disease Surveillance affected. Maps created using the Free and Open Source QGIS.

From September 2017, there was a great increase in the number of SYF epizootics in NHPs. This occurred from the increase in precipitation and temperature from August onwards and was followed by an increase in human cases from December 2017. The expansion of transmission to the eastern part of SP and those with low vaccine coverage was also identified (Figs. 1, 3, 5). Given this spatiotemporal sequence in the advance of the YF outbreak, the human cases and epizootics that occurred between September 2017 and 2019 were considered as the second SYF outbreak wave in SP. During the second wave, peaks in epizootics and human cases occurred in December 2017 and January 2018, respectively (Fig. 3).

In addition to the temporal occurrence of epizootic disease preceding human cases (Fig. 1), the outbreak also presented a distinct spatial pattern of occurrence. In the municipalities of the west SP region, epizootics without human cases were more frequent, whereas in the eastern region, the majority of municipalities with SYF presented both epizootics and human cases (Figs. 1, 4, 5).

**Figure 6:**
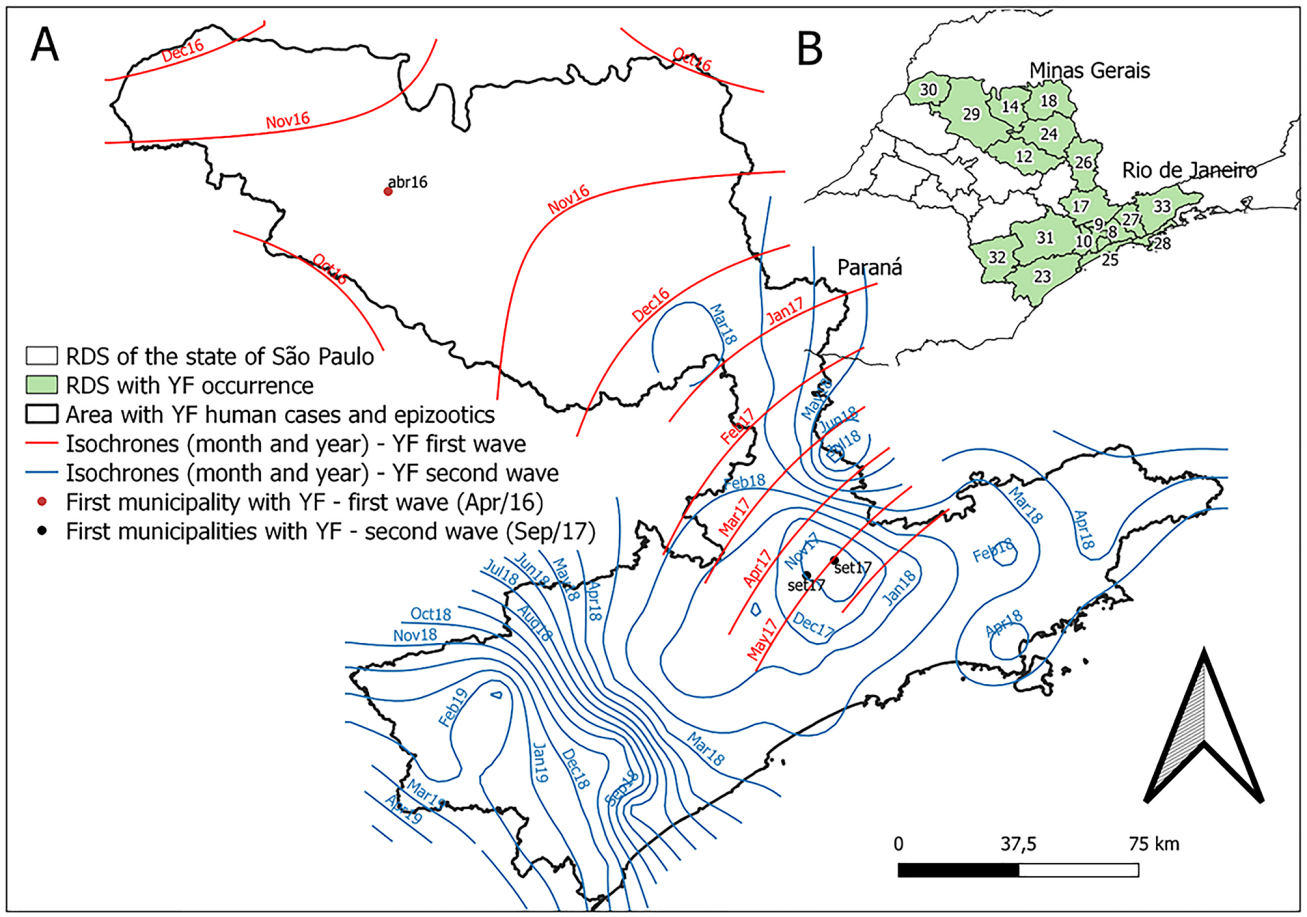
Mensal isochrones of the diffusion process of the yellow fever (YF) first and second waves in the state of São Paulo (**A**); Regional Disease Surveillance (RDS) with YF human cases and epizootics in the state of São Paulo and its bordering States (Minas Gerais, Paraná e Rio de Janeiro (**B**), Apr 2016 to September 2019. Maps created using the Free and Open Source QGIS.

The spatiotemporal advance of the first and second outbreak waves was confirmed through geostatistical modeling of the YF spread. The isochrones representing the first wave showed the advance from west to east (from São José do Rio Preto to Campinas). The second wave isochrones confirmed the path of the outbreak, from Campinas towards the municipalities bordering the states of Rio de Janeiro and Minas Gerais, the SP coast, and those bordering the state of Paraná (Fig. 6). Regarding the spreading speed, the first wave moved an average of 0.98 km per day. The second wave, in its displacement towards the states of Rio de Janeiro and Minas Gerais, had a speed of 0.86 km per day. In its displacement towards the state of Paraná, it presented two distinct speeds: 0.99 km per day to Sorocaba RDS (until February 2018) and 0.47 km per day to Registro and Itapeva RDS (from February 2018 onwards).

In 2016 (Fig. 1A), the YF virus transmission occurred in an area with high vaccine coverage where epizootic disease was the main occurrence. In 2017 and 2018, the transmission showed its greatest intensity and generally occurred in areas with vaccine coverage below 10%, manifesting both as epizootics and as human cases (Figs. 1B,C, 3). In 2019, the YF transmission became less intense than that of the previous 2 years and generally occurred in areas with vaccine coverage between 10 and 50% in 2018 (Figs. 1D, 3). The map analysis in Fig. 1 and the graph in Fig. 3 reveal that, at least in 2017 and 2018, previous levels of vaccination coverage were insufficient in preventing or minimizing the YF virus transmission.

## Discussion

The SYF outbreak that occurred in Brazil from 2016 onwards reached states in the southeast, northeast, and south. It became the most important outbreak in recent decades due to the large number of cases and deaths in humans and NHPs, reaching areas with high population densities and low vaccination coverage^5,12,19,20^. In SP, this process started in a region with high vaccination coverage and progressed to areas with low coverage and/or without vaccination recommendations, with high population densities^11,12^. The first region with these characteristics to be affected by the outbreak was Campinas^12^ and the period resulting from this advance was considered the first wave. In the second half of 2017, SYF advanced to the east, south, southeast, and northeast regions of SP, characterizing the second wave.

Hill et al.^4^ analyzed only the SYF epizootics that occurred in SP between 2016 and 2018 and divided this process into three phases. Although this makes more sense for the epizootic disease analysis, considering the outbreak process in two waves made it possible to adequately capture the spatiotemporal process of SYF diffusion and to characterize it by contagion. Moreover, this characterization allowed a discussion on the relevance of the vaccine strategy adopted by SES-SP, especially during the second wave. The Ministry of Health, in view of the YF outbreak in areas without recommendation and/or low vaccination coverage, advocated the expansion of vaccination based on calculations of affected area/expanded area, with the municipality as smallest unit^17^. Until mid-2017, this strategy was an adequate response. However, it became demonstrably insufficient when the virus reached populous areas with low coverage and/or without a vaccination recommendation, both in SP^12^ and in other southeastern Brazilian states^21^.

In SP, the advancement of YF within a period of less than six months from areas with high vaccination coverage to populous areas with low coverage or without vaccination recommendations triggered a public health crisis that generated panic in the population. At that time, there were insufficient time and vaccine doses to serve the entire population at risk^21^. Hence, SES-SP identified the priority vaccination areas based on the analysis of virus circulation in NHPs at forest fragments, which comprise functional ecological corridors for viral dispersion, and were therefore demarcated and used to select areas where populations were most exposed to YF risk. Instead of considering the entire municipal territory with the same risk level, the new strategy sought to identify priority intra-municipal areas for vaccination, considering the speed of dispersion^16^. Moreover, this strategy, plus the use of single doses^1^ and fractional doses^22^, allowed reasonable equation, in space–time, of the demand for vaccines in a large population contingent with an insufficient supply of the immunobiological product. It should be noted that this strategy had already been recommended by the WHO in outbreaks within the African continent in 2015/2016^21,23^.

Our results demonstrated that the YF virus dispersion in SP was caused by the outbreak process of territorial spread by contagion; therefore, the mosquito vectors and NHPs could act as a route of viral amplification and further transmission on epizootic waves. In this situation, there was a cadence in the spatiotemporal pattern of viral dispersion through contiguous and nearby areas. This also shows the appropriateness of the vaccination strategy adopted by SES-SP, which allowed the population to receive the vaccine at least two months before the establishment of on-site transmission risk^15^. A spatiotemporal process of sequential spreading was observed, wherein municipalities located at shorter distances from the areas with YF virus transmission were more likely to be affected first^7,8^. Although our analyses are limited by the fact that we used the municipality as a spatial study unit, this spreading pattern can also be observed at the intra-municipal level.

The speed of the SYF dispersion that we obtained for the first and second waves, disregarding the RDSs of Registro and Itapeva, were similar to those reported by Hill et al.^4^. Through phytogeographic analysis of YF genomes in NHPs, they reported spreading speeds of around 1.0 km per day. Notably, they took into account epizootics that occurred in SP up to February 2018, and the occurrences of human cases and epizootics in the RDS of Registro and Itapeva were recorded from February 2018 onwards. The differences in the speed of viral dispersion between these two RDSs and the rest of SP during the second wave may be related to the greater vegetation cover and forest preservation of these areas, which can cause a dilution effect, as already demonstrated for other vectorborne diseases^24^. The spread of the SYF outbreak by contagion in most of SP, with a speed of approximately 1.0 km per day, opened a spatiotemporal window of opportunity for the vaccine to arrive before the virus^15^, avoiding or minimizing the occurrence of human cases and deaths. Since 2019, this outbreak has advanced to the southern region of the country and has reached the states of Paraná and Santa Catarina, and is still ongoing. This generated an emergency situation similar to that in SP, and the same vaccination strategy adopted in SP has been applied in this region^16,18,20^.

Our findings (Fig. 2) showed important differences in human cases and epizootics that occurred in SP during the second wave, reflecting the degree to which municipalities have adopted the vaccination strategy advocated by the SES-SP. If this is true, what happened in Mairiporã and Atibaia (37% of the total human cases between 2016 and 2019) could also have occurred in municipalities such as Jundiaí, Bragança Paulista, Itapecerica da Serra, Pinhalzinho, Louveira, and São Paulo. If the YF vaccination strategy had not been adapted for the emergency situation during the second wave, we could have had a worse outcome than that observed. These possible scenarios could be the subject of future studies.

At the end of 2016 and 2017, the detection of YF epizootics in NHPs anticipated the notification of human cases by two and three months, respectively. This result is expected, since the seasonality observed among NHPs in Brazil differs from that observed in human cases. In primates, circulation is generally detected in September, whereas in humans, circulation is usually observed in December, with the detection of cases among non-immunized people and those exposed to the virus^17,25^. This also highlighted the importance of the NHP epizootic surveillance strategy, aimed at the early detection of the circulation of the YF virus while still in the enzootic cycle^4,26^. However, despite the heavy investment of SES-SP in making municipalities sensitive to detection of NHP mortality, the detection of epizootic diseases is still marked by a strong reporting bias^26^.

One of the causes for the anticipation of epizootics in relation to human cases can be explained by seasonality of the precipitation in SP during the year. From the middle to the end of autumn, depending on the year, the total rain decreased and registered monthly values that were increasingly smaller. This becomes reversed in the beginning of spring, when an increase in the precipitated volume begins to be registered throughout SP^27,28^. To increase YF transmission, mosquito vectors need to be found in large quantities, and one of the determining factors for the proliferation of mosquitoes is the level of precipitation, as this allows the accumulation of water in reservoirs and the hollows of trees^29^.

Another important factor is the rise in temperatures from the end of winter and the beginning of spring. Temperature increases accelerate the time for larval development of the vectors^29^ and reduces the extrinsic incubation period of the virus^30^. Precipitation and temperature directly influence the mosquito's life cycle and viral replication^31^, hence their increase is an optimal scenario for the proliferation of YF vectors and for the increased occurrence of epizootic diseases in SP^29^. By contrast, the increase in the probability of detecting human SYF cases in December may be related to the greater degree of exposure among unvaccinated people due, among other issues, to tourism, and to the fact that this occurs simultaneously with the transmission of the YF virus sustained by NHPs.

The occurrence of SYF outbreaks in regions with high population density and without adequate vaccine coverage represents a risk of YF reintroduction in urban areas. Even with the vaccination campaigns carried out so far, a large part of the Brazilian population has not yet been immunized^5^. UYF has been absent in Brazil since 1942, and in human cases that have occurred so far, there has been no epidemiological link with a possible urban cycle^20^ or involvement of its main urban vector, *Ae. aegypti*, in viral transmission^3^. However, this risk is increased by this vector’s presence in almost all Brazilian municipalities^32^. Another source of concern for the re-urbanization of YF in Brazil is the presence of *Ae. albopictus*. This mosquito reportedly transmits the YF virus in the laboratory setting and has already been found naturally infected by this virus in the city of Minas Gerais^20,33^. Present in both urban areas and the rural and forest areas of the country, this mosquito could be a link between the sylvatic and urban forms of the disease^3,34^. Efforts must be made to prevent the occurrence of UYF epidemics, as could constitute a major public health issue. Among the measures that can be adopted, the most urgent are investments in the production of vaccines, vaccination of the entire Brazilian population, and the development of effective measures to control *Ae. aegypti*^1,21,35^.

This study has several limitations, such as the use of secondary surveillance data, which are subject to both notification and underreporting errors. Examples of these problems are the need to eliminate three municipalities from wave modeling and the NHP epizootic underreport. The unavailability of the exact probable site of infection for human cases and epizootics, as well as their occurrence dates, obliged us to consider the centroids of the municipalities and the months of the year. These limitations provide a partial view of the outbreak and did not allow us to investigate, for example, the characteristics of the places where the cases and epizootics occurred. Another limitation was that vaccination coverage was based only on vaccination data for children under 5 years of age.

However, this study has strengths that contribute to its internal and external validity. Among them are the use of both information about sylvatic human cases, as well as epizootics, to investigate the outbreak process. Another strong point was the use of kriging geostatistics to assess the spread of SYF. This is a spatiotemporal process, and the use of kriging allowed us to consider the autocorrelation of the phenomenon in space–time and numerically represent it throughout the SP area.

## Conclusions

Our results showed that the SYF outbreak, which occurred in SP between 2016 and 2019, can be characterized as a contagion diffusion process. This disease, with a few exceptions, did not exhibit spatial jumps between municipalities, and its spatiotemporal dispersion occurred from the affected municipalities to those located in the immediate vicinity. Municipalities located at shorter distances from those with YF virus transmission were more likely to be affected first. These results indicate that mosquitoes and NHPs are a route of viral transmission in epizootic waves. This process showed general spreading speeds of approximately 1.0 km per day. Considering that predicting the spatiotemporal risk of the SYF occurrence is challenging, the strategy used by SES-SP to identify priority areas for vaccination and to anticipate the arrival of the virus was appropriate, at least in the short and medium terms. Moreover, the validity of this initiative is directly related to the outbreak process occurring through diffusion by contagion, wherein the vector and NHPs were responsible for viral dissemination. It is noteworthy that this strategy probably prevented the SYF outbreak in SP from resulting in a higher magnitude of human cases and deaths than that observed.

## Methods

This ecological and descriptive study was conducted in SP (Fig. 1) between 2016 and 2019. We included all YF autochthonous human cases and confirmed epizootics among NHPs in the state during the period considered. An epizootic event might be only one dead animal or few dead animals found in the same site, since the NHP species of importance in YF surveillance live in group and may be affected in the same period of time.

SP has an area of 248,000 km^2^ and is divided into 645 municipalities with a population of approximately 41 million. It is the most populous state in Brazil and has the second highest Human Development Index (0.783)^36^. Its climate is predominantly tropical, with periods of high temperatures in summer, dry weather in winter, and a high frequency of precipitation in the coastal region. Epidemiological surveillance actions in SP are coordinated by the Epidemiological Surveillance Center “Professor Alexandre Vranjac" of SES-SP (CVE), and developed regionally by the 28 RDSs (Fig. 1).

Data from autochthonous human cases and epizootics were obtained from the weekly YF Epidemiological Bulletins of CVE^14^. CVE investigated all human cases and mosquito specimens were collected. Since all mosquito species found in infection sites were indigenous sylvatic species, such as *Haemagogus* sp. and *Sabethes* sp., all cases were classified as SYF. On the other hand, during the studied period, no *Ae. aegypti* was ever found infected or reported as being a likely vector for any of the human cases, therefore no UYF was confirmed^14^.

Human cases were considered according to the month of symptoms onset, whereas the NHP epizootics were considered according to the month of notification. The data on vaccination coverage against YF among the municipalities were from 2015 to 2018, obtained from the National Immunization Program^37^. Since these data only contained coverage information among children under 5 year of age, they were used as a proxy for the entire population’s vaccine coverage. Data from precipitation and mean temperature of SP, by month, was obtained on the Integrated Agrometeorological Information Center (CIIAGRO) of the State Department of Agriculture and Supply of São Paulo.

Between 2016 and 2019, the number of confirmed SYF autochthonous human cases, deaths, and epizootics was computed according to the probable infection site, which refers to the municipality where the YF infection probably occurred. For each municipality with SYF autochthonous case(s) and/or epizootic(s) between 2016 and 2019, the month and year of the first occurrence(s) were recorded. Thus, the occurrence of human cases and epizootics was jointly considered as a proxy for the month of initiation of the YF transmission occurrence in a given municipality.

We obtained the total number of autochthonous human cases, deaths, and NHP epizootics, as well as the fatality rate of SYF for SP between 2016 and 2019. The numbers of SYF human cases and epizootics were attributed to the centroids of the municipalities according to months and years. Thematic maps were produced for the municipalities, with information about the occurrence of human only cases, epizootics, or both for a given year, and on the vaccination coverage of the previous year. This kind of data presentation was adopted because we considered 1-year coverage as a gold standard protection measure for preventing the occurrence of SYF human cases in the following year.

The number of human cases and NHP epizootics was calculated with respect to the month to present the temporal evolution of YF, characterize its seasonal behavior in relation to precipitation and mean temperature, and identify possible waves of development of this process. For each wave identified, we constructed a database containing the following information for each municipality with the autochthonous SYF transmission: Cartesian coordinates (in the Albers flat coordinate reference system) of the municipality centroids, and the month and year of the first occurrence of the SYF autochthonous human case and/or epizootic. Once constructed, the data was placed in increasing order of months and years, and we created a variable called space–time evolution to represent the evolution of the human cases and/or epizootics. At the municipality corresponding to the first month of occurrence, the value one was assigned; during the second month, the value two was assigned, and so on.

The SYF propagation is borne by epizootic events and human beings are accidentally infected during the process. During the propagation, the epizootics increase the number of infected mosquitos what increases the number of human cases, creating an epidemic shadow. Thus, we used the data of epizootics and human cases to compound the dataset of the analyses, since both are temporally and spatially connected. To refer to this process, we used the term SYF outbreak, representing the combination of epizootic and epidemic events.

For the characterization of the SYF diffusion in SP and the identification of its type, we chose a geostatistical interpolation technique capable of representing the spatiotemporal evolution of this outbreak process. The ordinary kriging technique was therefore used to interpolate the spatiotemporal evolution of the outbreak in each wave. This technique requires that the variable to be interpolated have a normal distribution and stationary behavior. For this, the variable was modeled squarely according to the coordinates of the municipal centroids and the kriging was performed from the residues obtained, with a good approximation for the distribution of normal probability and for a stationary trend. This procedure allowed the spatiotemporal evolution of the SYF outbreak to have numerical representation on the entire surface of the study area in each of the identified waves, which allowed the construction of maps with an isochronous representation. With these lines, it was possible to identify the type of diffusion that occurred and observe the directions in which the outbreak evolved, beyond calculating the speeds of its dispersion in kilometers per month.

Three municipalities were excluded from the modeling of the outbreak waves. Santa Cruz do Rio Pardo, located in the RDS of Assis (Fig. 1B), was excluded from the analysis because only one human case of YF in January 2017 with no spatial or temporal correlation to any other cases and. At the time, epidemiological investigations were triggered to determine the circulation of the virus in the region; however, no other evidence of viral circulation^14^ was found. The municipalities of Cachoeira Paulista and Itupeva were also excluded from the analyses because the cases happened 1 or 2 years after the passage the second outbreak wave. Therefore these cases most likely represents a persistent occurrence of the virus in those areas (what can happens up to 3 years) and do not contribute with any piece of information to the analyses of direction and velocity of the propagation wave. Thematic maps were constructed using QGIS software version 3.8.1^38^. Ordinary kriging was performed using the geoR package (version 1.8.1)^39^ of the R software version 4.0.2^40^.

## Data Availability

The datasets generated during and/or analysed during the current study are available from the corresponding author on reasonable request.

## References

[1] Cavalcante KRLJ, Tauil PL (2017). Risk of re-emergence of urban yellow fever in Brazil. Epidemiol. Serv. Saude.

[2] Tauil PL (2010). Critical aspects of yellow fever control in Brazil. Rev. Saude Publ..

[3] Abreu FVSD, Ribeiro IP, Ferreira-de-Brito A, Santos AACD, Miranda RMD, Bonelly IDS, Lourenço-de-Oliveira R (2019). *Haemagogus leucocelaenus* and *Haemagogus janthinomys* are the primary vectors in the major yellow fever outbreak in Brazil, 2016–2018. Emerg. Microbes Infect..

[4] Hill SC, de Souza R, Thézé J, Claro I, Aguiar RS, Abade L, Faria NR (2020). Genomic surveillance of yellow fever virus epizootic in São Paulo, Brazil, 2016–2018. PLoS Pathog..

[5] Sacchetto L, Drumond BP, Han BA, Nogueira ML, Vasilakis N (2020). Re-emergence of yellow fever in the neotropics—quo vadis?. Emerg. Top. Life Sci..

[6] Catão RDC (2016). Expansão e Consolidação do Complexo Patogênico do Dengue no Estado de São Paulo: Difusão Espacial e Barreiras Geográficas.

[7] Haggett P (2020). The Geographical Structure of Epidemics.

[8] Cliff AD, Ord JK, Haggett P, Versey GR (1981). Spatial diffusion: an historical geography of epidemics in an island community. CUP Arch..

[9] Franco O (1969). História da febre amarela no Brasil [Internet]. Rio de Janeiro Ministério da Saúde Depart. Nacl. Endemias Rurais.

[10] Saad LDC, Barata RB (2016). Surtos de febre amarela no estado de São Paulo, 2000–2010. Epidemiol. Serv. Saude.

[11] MS. Ministério da Saúde. Secretaria de Vigilância em Saúde. Municípios com recomendação para vacinação contra febre amarela. (2015). https://portalarquivos2.saude.gov.br/images/pdf/2015/novembro/19/Lista-de-Municipios-ACRV-Febre-Amarela-Set-2015.pdf. Accessed 13 Mar 2021.

[12] Cunha MS, da Costa AC, de Azevedo Fernandes NCC, Guerra JM, Dos Santos FCP, Nogueira JS, de Souza RP (2019). Epizootics due to yellow fever virus in São Paulo State, Brazil: viral dissemination to new areas (2016–2017). Sci. Rep. UK.

[13] Rezende IMD, Sacchetto L, Munhoz de Mello É, Alves PA, Iani FCDM, Adelino TÉR, Drumond BP (2018). Persistence of Yellow fever virus outside the Amazon Basin, causing epidemics in Southeast Brazil, from 2016 to 2018. PLoS NTD.

[14] CVE-Centro de Vigilância Epidemiológica Professor Alexandre Vranjac. Boletim Epidemiológico. (2019). http://saude.sp.gov.br/cve-centro-de-vigilancia-epidemiologica-prof.-alexandre-vranjac/areas-de-vigilancia/doencas-de-transmissao-por-vetores-e-zoonoses/agravos/febre-amarela/boletim-epidemiologico. Accessed 11 Aug 2020.

[15] Fioravanti, C. H. O combate à febre amarela no estado de São Paulo: histórias, desafios e inovações. In *O Combate à Febre Amarela No Estado de São Paulo: Histórias, Desafios e Inovações*. Press 183–183 (2018).

[16] MS-Ministério da Saúde. Secretaria de Vigilância em Saúde. Saúde Brasil 2019: Uma análise da situação de saúde com enfoque nas doenças imunopreveníveis e na imunização. (2019a). https://portalarquivos2.saude.gov.br/images/pdf/2019/dezembro/05/Saude-Brasil-2019-imunizacao.pdf. Accessed on 30 Mar 2021.

[17] MS-Ministério da Saúde. Secretaria de Vigilância em Saúde. Guia de Vigilância Epidemiológica. (2019b). https://portalarquivos2.saude.gov.br/images/pdf/2019/junho/25/guia-vigilancia-saude-volume-unico-3ed.pdf. Accessed on 13 Mar 2021.

[18] MS - Ministério da Saúde. Secretaria de Vigilância em Saúde. Boletim Epidemiológico Boletim Epidemiológico 04. (2021). https://www.gov.br/saude/pt-br/media/pdf/2021/fevereiro/11/boletim_epidemiologico_svs_4.pdf. Accessed on 14 Apr 2021.

[19] Giovanetti M, de Mendonça MCL, Fonseca V, Mares-Guia MA, Fabri A, Xavier J, de Filippis AMB, Correction for Giovanetti (2020). Yellow fever virus reemergence and spread in Southeast Brazil, 2016–2019. Virol. J..

[20] Silva NIO, Sacchetto L, de Rezende IM, Trindade GDS, de LaBeaud AD, Thoisy B, Drumond BP (2020). Recent sylvatic yellow fever virus transmission in Brazil: the news from an old disease. Virol. J..

[21] Possas C, Lourenço-de-Oliveira R, Tauil PL, Pinheiro FDP, Pissinatti A, Cunha RVD, Homma A (2018). Yellow fever outbreak in Brazil: the puzzle of rapid viral spread and challenges for immunisation. Mem. I Oswaldo Cruz.

[22] Juan-Giner A, Kimathi D, Grantz KH, Hamaluba M, Kazooba P, Njuguna P, Grais RF (2021). Immunogenicity and safety of fractional doses of yellow fever vaccines: a randomised, double-blind, non-inferiority trial. Lancet.

[23] WHO-World Health Organization Department of Immunization, Vaccines and Biologicals. Fractional dose yellow fever vaccine as a dose-sparing option for outbreak response. Geneva, Word Health Organization, (2016). https://apps.who.int/iris/bitstream/handle/10665/246236/WHO-YF-SAGE-16.1-eng.pdf%3Bjsessionid%3DF4BD11CEBB8426D52C781267F9795A8A%3Fsequence%3D1. Accessed on May 2021.

[24] Keesing F, Holt RD, Ostfeld RS (2006). Effects of species diversity on disease risk. Ecol. Lett..

[25] Vasconcelos PFDC (2003). Febre amarela. Rev. Soc. Bras. Med. Trop..

[26] Romano APM, Ramos DG, Araújo FAA, Siqueira GAMD, Ribeiro MPD, Leal SG, Elkhoury ANMS (2011). Febre amarela no Brasil: recomendações para a vigilância, prevenção e controle. Epidemiol. Serv. Saude..

[27] Schröder R (1956). Distribuição e curso anual das precipitações no Estado de São Paulo. Bragantia.

[28] Galvani E, de Lima NGB, Alves RR (2012). Variabilidade e tendência das precipitações no litoral sul de São Paulo. Geonorte.

[29] Urbinatti PR, Menezes RMTD, Natal D (2007). Sazonalidade de Aedes albopictus em área protegida na cidade de São Paulo, Brasil. Rev. Saude Publ..

[30] Meade MS, Emch M (2010). Medical Geography.

[31] Torres RG, Moreira VM, Neves RA (2019). Análise da distribuição espacial dos casos de febre amarela no estado de Goiás, 2007–2017. Rev. Bras. Mil. Cien..

[32] Nunes MRT, Faria NR, de Vasconcelos JM, Golding N, Kraemer MU, de Oliveira LF, da Costa Vasconcelos PF (2015). Emergence and potential for spread of Chikungunya virus in Brazil. BMC Med..

[33] Faria NR, Kraemer MU, Hill SC, De Jesus JG, Aguiar RS, Iani FC, Pybus OG (2018). Genomic and epidemiological monitoring of yellow fever virus transmission potential. Science.

[34] Pereira dos Santos T, Roiz D, Santos de Abreu FV, Luz SLB, Santalucia M, Jiolle D, Paupy C (2018). Potential of Aedes albopictus as a bridge vector for enzootic pathogens at the urban-forest interface in Brazil. Emerg. Mic. Inf..

[35] Noronha TGD, Camacho LAB (2017). Controversies in the expansion of areas with routine yellow fever vaccination in Brazil. Cad. Saude Publ..

[36] IBGE-Instituto Brasileiro de Geografia e Estatística. Cidades e Estados. (2021). https://www.ibge.gov.br/cidades-e-estados/sp.html. Accessed 13 Mar 2021.

[37] DATASUS-Departamento de Informática do SUS. Imunizações. (2021). http://tabnet.datasus.gov.br/cgi/dhdat.exe?bd_pni/cpnibr.def. Accessed on 11 Aug 2021.

[38] QGIS-QGIS Geographic Information System. QGIS Association. (2020). http://www.qgis.org.

[39] Ribeiro-Jr, P. J., Diggle, P. J., Schlather, M., Bivand, R., Ripley, R. geoR: Analysis of Geostatistical Data. R package version 1.8-1. (2020). https://CRAN.R-project.org/package=geoR.

[40] R Core Team. *R: A Language and Environment for Statistical Computing*. R Foundation for Statistical Computing, Vienna, Austria. (2020). https://www.R-project.org/.

